# Effects of different iodine supplementation strategies on thyroid function in iodine-deficient pregnant women: a meta-analysis

**DOI:** 10.3389/fpubh.2026.1846475

**Published:** 2026-06-10

**Authors:** Yingying Lv, Baiming Jin, Jiazhuang Guo, Jingshu Bu, Chen Chen, Yunfeng Han, Zhiping Xie, Yuehui Jia, Siyuan Wan, Chunjing Zhang

**Affiliations:** 1 School of Public Health, Qiqihar Medical University, Qiqihar, Heilongjiang, China; 2 Postdoctoral Workstation, Research Institute of Medical and Pharmacy, Qiqihar Medical University, Qiqihar, Heilongjiang, China; 3 Department of Biochemistry and Molecular Biology, Qiqihar Medical University, Qiqihar, Heilongjiang, China

**Keywords:** iodine deficient, iodine supplementation, meta-analysis, pregnant women, thyroglobulin, thyroid function, thyroid volume

## Abstract

**Background:**

This study aimed to clarify the association between iodine supplementation and thyroid function indices, as well as secondary outcomes including thyroglobulin (Tg) and thyroid volume (TV), in iodine-deficient pregnant women, accounting for different supplementation regimens and trimesters.

**Methods:**

Chinese and English literature on iodine supplementation in iodine-deficient pregnant women and its associations with thyroid function indices, Tg, and TV was retrieved from PubMed, Medline, Embase, CNKI, WanFang, and WeiPu databases. Effect sizes were estimated using fixed- and random-effects models, reported as standardized mean difference (SMD), odds ratio (OR), and 95% confidence intervals (CIs).

**Results:**

Iodine supplementation was associated with reduced serum TSH and Tg levels in iodine-deficient pregnant women (TSH: SMD = −0.14, 95% CI: −0.23, −0.05; *Pz* = 0.002; Tg: SMD = −0.22, 95% CI: −0.32, −0.13; *Pz* < 0.001). Subgroup analyses showed significant TSH reductions in Asian populations, third trimester, daily supplementation, 200–300 μg/d doses, and potassium iodide use (*Pz* < 0.05). Tg was significantly reduced across all regions, iodine supplementation dosages and methods, second/third trimesters, and daily supplementation subgroups (*Pz* < 0.05). TV was significantly increased in the Europe subgroup and the until postpartum supplementation subgroup (*Pz* < 0.01). Egger's test suggested publication bias for TV, but results remained robust after trim-and-fill adjustment.

**Conclusion:**

Standardized iodine supplementation notably reduces serum TSH and Tg in iodine-deficient pregnant women, relieving gestational thyroid dysfunction and optimizing maternal thyroid health. Greater TSH improvements are observed in Asian populations, the third trimester, and those taking 200–300 μg potassium iodide daily.

**Systematic review registration:**

https://www.crd.york.ac.uk/PROSPERO/view/CRD420261323017, identifier: CRD420261323017.

## 1 Introduction

Iodine, as an essential trace element for the human body, is also a necessary raw material for the synthesis of thyroid hormones. Insufficient iodine intake can lead to a series of iodine deficiency disorders (IDD), such as compensatory thyroid hyperplasia and hypothyroidism. These diseases are also important public health issues that have attracted global attention (1). Since 1993, the World Health Organization (WHO) and the United Nations Children's Fund (UNICEF) have jointly implemented the Universal Salt Iodization (USI) strategy. This intervention has achieved remarkable results in improving the iodine nutrition status of the population, particularly in effectively optimizing the iodine deficiency status of pregnant women (2, 3).

Pregnant women are a high-risk group for iodine deficiency. During pregnancy, blood volume increases, glomerular filtration rate rises, and the demand for iodine by the fetus for growth and development continues to grow, making the iodine requirement for pregnant women significantly higher throughout the pregnancy stage compared to the non-pregnancy period (4). Globally, 72 countries have reported data on iodine nutrition among pregnant women, and 39 of them (approximately 54%) have inadequate iodine intake in this population (5). In Europe, nearly two-thirds of countries fail to meet the iodine nutrition standards for pregnant women. Widespread iodine deficiency in pregnant women has been documented in Australia, the United Kingdom, Spain, the Netherlands, the United States, and other nations (6). A United States study showed that pregnant women's iodine intake in the United States declined continuously from 2011 to 2020, with over 60% experiencing inadequate iodine nutrition between 2017 and 2020 (7). A 2020 assessment covering 31 provincial-level regions in China revealed that pregnant women overall had mild iodine deficiency, with an iodine deficiency rate of 51.6% and a moderate deficiency rate of 26.94%. The severity of iodine deficiency progressively increases as pregnancy advances (8). Overall, iodine deficiency in the mother during pregnancy can have adverse effects on the health of both the mother and the fetus: for the pregnant woman, it can significantly increase the risk of hypothyroidism and goiter; for the offspring, iodine deficiency not only hinders fetal growth and development but may also impair the level of infant intellectual development (9, 10). Therefore, optimizing iodine nutrition intake strategies for special populations is a crucial measure in the public health prevention and control system to reduce the prevalence of thyroid diseases among the population (11).

Maintaining adequate iodine nutrition during pregnancy can effectively improve the iodine nutritional status of pregnant women with iodine deficiency. However, there is still significant controversy in the existing research regarding the impact of strategies for iodine supplementation during pregnancy, such as the total duration of supplementation and the timing of its implementation, on thyroid function. Studies have shown that long-term iodine supplementation may increase the risk of thyroid dysfunction during pregnancy for pregnant women (12). A study conducted in mild iodine-deficient areas of Russia suggests that pregnant women who received iodine supplementation at a dose of 150–200 μg/d had significantly lower thyroid volumes (TV) compared to those who did not receive iodine supplementation or who started receiving it during the second trimester (13). At the same time, there are also studies suggesting that iodine supplementation did not have a significant effect on the thyroid volume of pregnant women with iodine deficiency (14). More importantly, in the existing related studies, the impact of different iodine supplementation stages during pregnancy on the thyroid function of pregnant women and related outcome indicators such as thyroglobulin (Tg) has not yet reached a unified understanding (15). Studies have shown that adequate iodine nutrition during pregnancy can improve thyroid function and reduce the risk of abnormally elevated Tg levels (16). However, other studies have shown that supplementing iodine during early pregnancy may lead to thyroid dysfunction in pregnant women with mild iodine deficiency (17). The use of iodine-containing supplements during different stages of pregnancy, or even before conception, may exert varying effects on the levels of thyroid-stimulating hormone (TSH), free triiodothyronine (FT_3_), and free tetraiodothyronine (FT_4_) in pregnant women (18).

Currently, the iodine nutrition intervention strategy for pregnant women has globally established a comprehensive model primarily relying on iodized salt, supplemented by iodine supplements. However, there are still variations in the methods, dosages, durations of iodine supplementation, as well as its adaptability to different populations (19). In terms of iodine supplementation methods, USI remains the most fundamental and widely implemented public health measure, which can achieve continuous and low-dose iodine intake at the population level (20). Iodine supplements (such as potassium iodide and potassium iodate tablets) are mostly used in areas with iodine deficiency or for high-risk pregnant women as a form of intervention. Regarding the dosage of iodine supplementation, the WHO recommends a daily iodine intake of 250 μg for pregnant women, while the Institute of Medicine (IOM) of the United States advises a pregnancy iodine intake of 220 μg/d, but there are differences in the recommended supplement doses among different countries (21). Regarding the duration of iodine supplementation, the prevailing view is that continuous iodine supplementation should be carried out from before pregnancy to throughout the entire pregnancy. Especially during the second and third trimesters, the iodine requirement significantly increases, which is a crucial intervention period. However, in some regions, there are still cases where only iodine supplementation is done in the early pregnancy and then interrupted in the middle and late stages, which may affect the stability of thyroid function (22). Therefore, the adaptability of intervention strategies is particularly important for different groups of pregnant women.

In summary, although there have been relevant studies exploring the impact of iodine supplementation on thyroid function and secondary outcomes such as Tg and TV indicators in pregnant women (12–16), existing research has mostly failed to focus on the differentiated effects of different pregnancy stages, iodine supplementation methods, durations, and dosages. The relevant research evidence still needs further refinement and integration. Based on this, this study will systematically review the existing related research, focusing on different iodine supplementation methods, different iodine supplementation doses, and different pregnancy stages, to clarify the relationship between iodine supplementation for pregnant women with iodine deficiency and thyroid function, as well as the secondary outcome indicators Tg and TV of iodine supplementation. This will provide more targeted evidence-based medical evidence for the clinical standardization of iodine supplementation plans for pregnant women with iodine deficiency and for ensuring the health of mothers and infants.

## 2 Materials and methods

### 2.1 Article retrieval methods and strategies

All included literature underwent stringent evaluation in accordance with the Preferred Reporting Items for Systematic Reviews and Meta-Analyses (PRISMA 2020) statement, with a subsequent meta-analysis being performed (23). A systematic search was conducted across the electronic databases of PubMed, Medline, and Embase to identify international and domestic studies exploring the associations between iodine supplementation, thyroid function-related indicators, and the secondary outcome indicators Tg and TV in pregnant women. Concurrently, a targeted search for Chinese-language publications was undertaken in the China National Knowledge Infrastructure (CNKI), WanFang Database, and WeiPu Chinese Science and Technology Journal Database. The databases were searched for articles published before March 10, 2026. The searches were carried out with the help of a combination of MeSH (medical subject headings) terms and key terms. The following search terms were used: (“pregnancy” OR “pregnant women” OR “maternal” OR “gestational”) AND (“urine iodine” OR “iodine status” OR “urine iodine concentration”) AND (“iodine supplementation” OR “iodine intervention”) AND (“thyroid function tests” OR “thyroid gland function tests” OR “FT_3_” OR “free triiodothyronine” OR “FT_4_” OR “free tetraiodothyronine” OR “TSH” OR “thyroid-stimulating hormone” OR “TPOAb” OR “thyroperoxidase antibody” OR “TgAb” OR “thyroglobulin antibody”). This meta-analysis was registered at https://www.crd.york.ac.uk/PROSPERO/view/CRD420261323017.

### 2.2 Inclusion and exclusion criteria

In this study, the definition of iodine deficiency in pregnant women is based on the standards of WHO/UNICEF/ICCIDD, which stipulate that a maternal urinary iodine concentration (UIC) of < 150 μg/L denotes iodine deficiency. A urinary iodine concentration threshold of 150–249 μg/L is used to define adequate iodine nutritional status in pregnant women (19).

Inclusion criteria: (i) Randomized controlled trials or prospective cohort studies that can reflect the association between iodine supplementation in iodine-deficient pregnant women and thyroid function, together with secondary outcome indices such as Tg and TV; (ii) Case-control study design; (iii) Case group: pregnant women who were iodine-deficient before commencing iodine supplementation (UIC < 150 μg/L, or urinary iodine-to-creatinine ratio < 150 μg/g) (19). The iodine supplementation protocol should have clear intervention measures, initiation time, total duration and dosage; control group: pregnant women who received iodine supplementation placebo or no iodine supplementation; (iv) Complete and analyzable data that can be used or converted for calculation; (v) Only articles published in English or Chinese will be considered.

Exclusion criteria: (i) Absence of a control group; (ii) Duplicate publications, low-quality literature and literature with missing data; (iii) Literature that cannot provide original data, such as reviews, meta-analyses, animal experiments and conference abstracts.

### 2.3 Data extraction and quality assessment

Initially, we screen the article titles and abstracts. If no screening results can be obtained, we proceed to read the full text. Subsequently, we include eligible articles and exclude those that do not meet the criteria. Lastly, for the articles that meet the inclusion criteria, we extract key information, including the first author's name, publication year, country, gestation, urinary iodine status, iodine supplementation method, total duration of iodine supplementation, iodine supplementation dose, thyroid function status, Tg levels, TV levels, and other primary outcome indicators. Notably, two researchers independently completed literature screening and data extraction throughout the above procedures. Any discrepancies were first resolved through cross discussion and negotiation. If no consensus was reached, a third researcher made the final judgment to ensure the accuracy and reliability of the research data. Meanwhile, the methodological quality of the included articles was assessed using the cross-sectional study evaluation tool recommended by the Agency for Healthcare Research and Quality (AHRQ) (24). If the answer was “no” or “unclear”, the score for that item was “0”; if the answer was “yes”, the score for that item was “1”. The quality scores for the articles were as follows: low quality = 0–3 points; moderate quality = 4–7 points; high quality = 8–11 points.

### 2.4 Statistical analysis

For continuous outcome measures in this study, the standardized mean difference (SMD) with its 95% confidence interval (CI) was used to describe the effect size (25–27). For dichotomous variables, the odds ratio (OR) with the corresponding 95% CI was employed as the effect statistic. Heterogeneity was assessed using the *I*^2^ statistic, and the model for pooling effects was selected based on the test results: a fixed-effects model was adopted if *I*^2^ < 50%, otherwise a random-effects model was used. To identify the sources of heterogeneity, subgroup analyses were performed stratified by country, gestational period, total duration of iodine supplementation, iodine supplementation dose, and iodine supplementation method. In the meta-analysis, a two-tailed *P* value (*Pz*) < 0.05 was considered statistically significant. Publication bias was evaluated using Egger's test (28), with a *P* value < 0.10 indicating statistically significant publication bias. All statistical analyses were conducted using STATA software 15.0 (Stata Corporation, College Station, TX, USA).

## 3 Results

In this study, we primarily extracted the main outcome indicators related to thyroid function, including FT_3_, FT_4_, TSH, as well as thyroid function-related antibodies TPOAb and TgAb, before and after iodine supplementation for iodine-deficient pregnant women. Additionally, secondary outcome indicators closely related to iodine supplementation, including Tg and TV, were also included. The results of this meta-analysis are as follows.

### 3.1 Study characteristics

**Figure 1:**
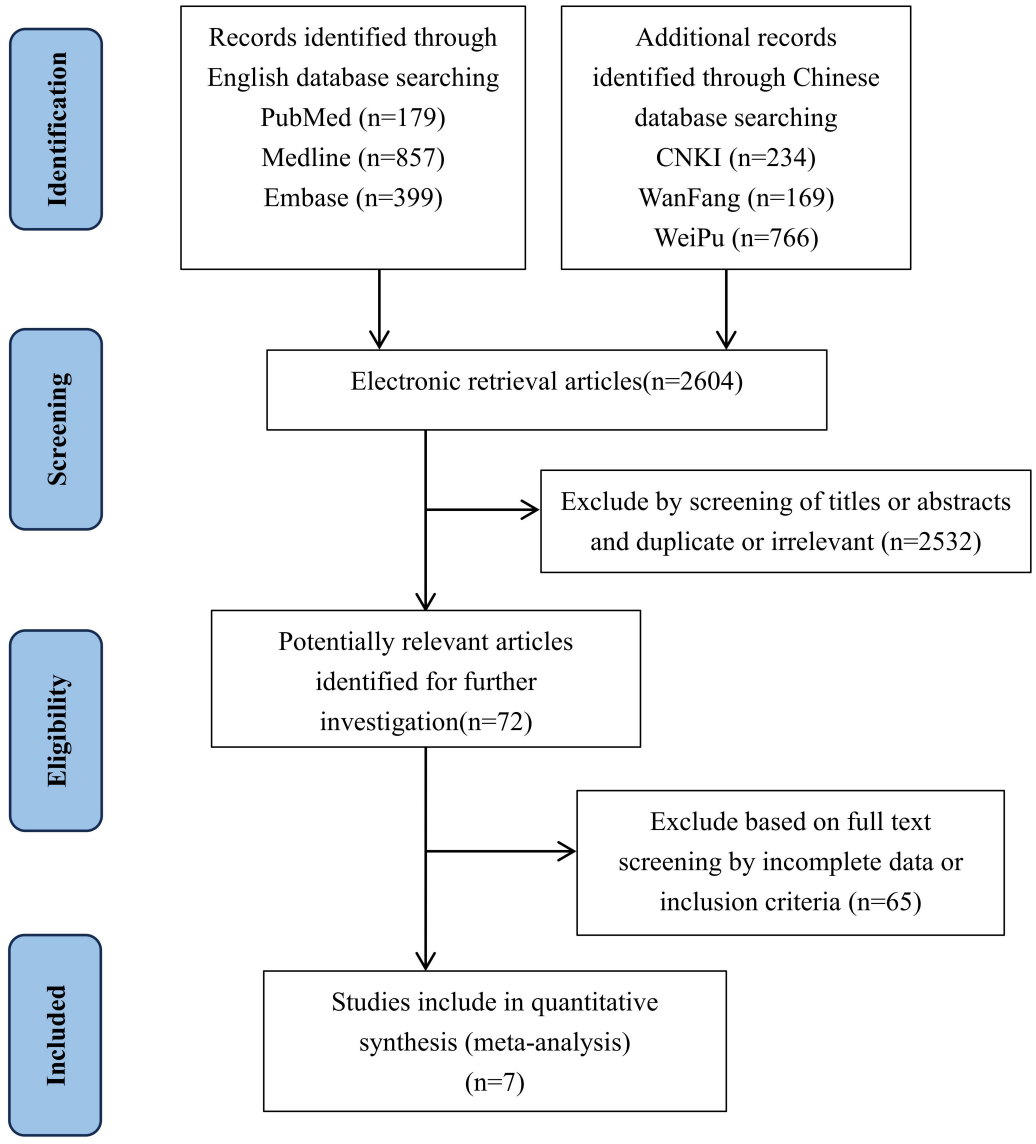
Flow diagram of study selection for review.

A total of 2604 articles were retrieved through the database, and ultimately, 7 studies met the inclusion criteria, encompassing a total of 10 studies (Figure 1). The main characteristic data of all included studies are presented in Tables 1, 2. The study encompassed 2,775 subjects from 7 countries. It included 5 randomized controlled trials (29–33) and 2 observational studies (34, 35), with 2 studies originating from Asia (31, 35) and 5 from Europe (29, 30, 32–34). Gestational periods were categorized into first trimester (33, 34), second trimester (29, 31, 32), third trimester (29–32), and overall trimester (35). Iodine supplementation methods for iodine-deficient pregnant women were divided into potassium iodide (31–34) and iodine-containing vitamins or/and minerals (29, 30, 35). Total duration of iodine supplementation was classified as at least 3 months (35), daily supplementation (29–31, 34), and iodine supplementation from pregnancy to postpartum (32, 33). The range of iodine supplementation dosage was 150–300 μg/d (29–35).

**Table 1:** Characteristics and data of 7 articles (FT_3_, FT_4_, TSH, Tg, and TV).

No. author	Year	Country	Gestation	BUIL		ISM	TDIS	ISD (μg/d)	FT_3_ ($\bar{\text{X}}\pm \text{S}$)	FT_4_ ($\bar{\text{X}}\pm \text{S}$)	TSH ($\bar{\text{X}}\pm \text{S}$)	Tg ($\bar{\text{X}}\pm \text{S}$)	TV ($\bar{\text{X}}\pm \text{S}$)	Quality evaluation
				Case	Control									
(34) Lopes-Pereira	2024	Portugal	First trimester	90 (*n =* 48) (μg/L)	66 (*n =* 37)	Potassium iodide	Everyday	150–200	(3.27 ± 0.33)^†^ vs. (3.34 ± 0.37)^‡^ (pg/ml)	(1.10 ± 0.11)^†^ vs. (1.10 ± 0.10)^‡^ (ng/dL)	(1.52 ± 0.89)^†^ vs. (1.43 ± 0.77)^‡^ (mUI/L)	(27.34 ± 21.09)^†^ vs. (30.48 ± 25.68)^‡^ (μg/L)	(13.20 ± 3.70)^†^ vs. (11.90 ± 4.10)^‡^ (*cm*^3^)	8
(35) Zhao	2004	China	Overall trimester	< 150 (*n =* 55) (μg/L)	< 150 (*n =* 38)	Multivitamin with Iodine	At least three months	150	(4.05 ± 2.57)^†^ vs. (3.91 ± 2.99)^‡^ (pmol/L)	(18.61 ± 10.70)^†^ vs. (21.63 ± 13.14)^‡^ (pmol/L)	(1.20 ± 0.54)^†^ vs. (1.52 ± 0.66)^‡^ (mU/L)	(31.11 ± 8.81)^†^ vs. (36.19 ± 9.63)^‡^ (ng/ml)	—	9
(29) Manousou	2021a	Sweden	Second trimester	110 (*n =* 81) (μg/L)	111 (*n =* 79)	Multivitamin with Iodine	Everyday	150	—	(12.65 ± 0.76)^†^ vs. (12.00 ± 1.51)^‡^ (pmol/L)	(1.63 ± 0.60)^†^ vs. (1.64 ± 0.68)^‡^ (mIE/L)	(16.71 ± 12.09)^†^ vs. (21.76 ± 14.37)^‡^ (μg/L)	—	8
	2021b	Sweden	Third trimester	110 (*n =* 65) (μg/L)	110 (*n =* 70)	Multivitamin with Iodine	Everyday	150	—	(12.00 ± 1.52)^†^ vs. (12.00 ± 1.51)^‡^ (pmol/L)	(1.86 ± 0.83)^†^ vs. (2.03 ± 1.06)^‡^ (mIE/L)	(24.12 ± 16.67)^†^ vs. (33.82 ± 24.24)^‡^ (μg/L)	—	
(30) NØHR	2000	Denmark	Third trimester	52 (*n =* 42) (μg/L)	51 (*n =* 24)	Iodine vitamin and mineral	Everyday	150	(4.89 ± 0.86)^†^ vs. (5.10 ± 0.86)^‡^ (pmol/L)	(10.99 ± 2.30)^†^ vs. (10.87 ± 2.76)^‡^ (pmol/L)	(1.59 ± 0.75)^†^ vs. (1.70 ± 0.86)^‡^ (mU/L)	(13.50 ± 12.67)^†^ vs. (20.44 ± 19.94)^‡^ (μg/L)	—	7
(31) Gowachirapant	2017a	India Thailand	Second trimester	135 (*n =* 291) (μg/L)	125 (*n =* 274)	Potassium iodide	Everyday	200	(3.61 ± 0.79)^†^ vs. (3.60 ± 0.89)^‡^ (ng/L)	(0.85 ± 0.13)^†^ vs. (0.86 ± 0.13)^‡^ (ng/dL)	(1.37 ± 0.75)^†^ vs. (1.44 ± 0.82)^‡^ (mIU/L)	(8.37 ± 6.84)^†^ vs. (9.20 ± 6.99)^‡^ (μg/L)	(7.36 ± 2.27)^†^ vs. (7.30 ± 2.52)^‡^ (ml)	9
	2017b	India Thailand	Third trimester	135 (*n =* 280) (μg/L)	125 (*n =* 299)	Potassium iodide	Everyday	200	(3.60 ± 0.78)^†^ vs. (3.60 ± 0.91)^‡^ (ng/L)	(0.83 ± 0.15)^†^ vs. (0.84 ± 0.14)^‡^ (ng/dL)	(1.34 ± 0.67)^†^ vs. (1.46 ± 0.67)^‡^ (mIU/L)	(9.16 ± 7.25)^†^ vs. (10.58 ± 8.84)^‡^ (μg/L)	(7.29 ± 2.20)^†^ vs. (7.33 ± 2.14)^‡^ (ml)	
(32) Censi	2019a	Italy	Second trimester	55.37 (*n =* 50) (ug/g)	50.98 (*n =* 38)	Potassium iodide	Until 8 weeks after delivery	225	(4.32 ± 0.23)^†^ vs. (4.10 ± 0.24)^‡^ (pmol/L)	(12.81 ± 1.24)^†^ vs. (12.98 ± 0.89)^‡^ (pmol/L)	(1.58 ± 0.56)^†^ vs. (1.58 ± 0.32)^‡^ (mIU/L)	(7.67 ± 4.85)^†^ vs. (8.13 ± 3.58)^‡^ (ng/ml)	(12.04 ± 3.93)^†^ vs. (10.42 ± 2.76)^‡^ (*mm*^3^)	8
	2019b	Italy	Third trimester	55.37 (*n =* 40) (ug/g)	50.98 (*n =* 33)	Potassium iodide	Until 8 weeks after delivery	225	(4.24 ± 0.28)^†^ vs. (4.28 ± 0.33)^‡^ (pmol/L)	(12.76 ± 1.11)^†^ vs. (12.99 ± 0.57)^‡^ (pmol/L)	(1.48 ± 0.51)^†^ vs. (1.87 ± 0.37)^‡^ (mIU/L)	(6.48 ± 3.28)^†^ vs. (10.29 ± 8.89)^‡^ (ng/ml)	(11.68 ± 2.31)^†^ vs. (10.95 ± 1.40)^‡^ (*mm*^3^)	
(33) Liesenkötter	1996	Germany	First trimester	49.2 (*n =* 38) (μg/g)	54.9 (*n =* 70)	Potassium iodide	Pregnancy and postpartum	300	—	—	(0.84 ± 1.10)^†^ vs. (0.70 ± 0.90)^‡^ (mU/L)	(16.50 ± 18.50)^†^ vs. (16.60 ± 19.20)^‡^ (μg/L)	–	8

BUIL, baseline urinary iodine level; ISM, iodine supplementation methods; TDIS, total duration of iodine supplementation; ISD, iodine supplementation dosage; FT_3_, free triiodothyronine; FT_4_, free thyroxine; TSH, thyroid stimulating hormone; Tg, thyroglobulin; TV, thyroid volumes. a, b = different studies in the same article; ^†^values refer to case group; ^‡^values refer to control group.

**Table 2:** Characteristics and data of 3 articles (TgAb and TPOAb).

No. author	Year	Country	BUIL (μg/L)		ISM	TDIS	ISD (μg/d)	Abnormal TgAb (%)	Abnormal TPOAb [($\bar{\text{X}}\pm \text{S}$) *n*]	Quality evaluation
			Case	Control						
(35) Zhao	2004	China	<150 (*n =* 55)	<150 (*n =* 38)	Multivitamin with Iodine	At least 3 months	150	3.63^†^ vs. 0.00^‡^	—	9
(30) NØHR	2000	Denmark	52 (*n =* 42)	51 (*n =* 24)	Iodine vitamin and mineral	Everyday	150	41.00^†^ vs. 54.00^‡^	150.36 ± 199.57)^†^ vs. (152.56 ± 209.62)^‡^ (*n =* 42) (*n =* 24) (U/ml)	7
(31) Gowachirapant	2017a	India Thailand	135 (*n =* 291)	125 (*n =* 274)	Potassium iodide	Everyday	200	—	13.86 ± 7.00)^†^ vs. (14.27 ± 7.75)^‡^ (*n =* 299) (*n =* 291) (IU/ml)	9
	2017b	India Thailand	135 (*n =* 280)	125 (*n =* 299)	Potassium iodide	Everyday	200	—	13.44 ± 6.56)^†^ vs. (13.05 ± 6.48)^‡^ (*n =* 276) (*n =* 295) (IU/ml)	9

BUIL, baseline urinary iodine level; ISM, iodine supplementation methods; TDIS, total duration of iodine supplementation; ISD, iodine supplementation dosage; TPOAb, thyroid peroxidase antibody; TgAb, thyroglobulin antibody; Abnormal TPOAb/TgAb, the antibody levels exceeded the upper limit of the reference range. a, b =different studies in the same article; ^†^values refer to case group; ^‡^values refer to control group.

### 3.2 Iodine supplementation in iodine-deficient pregnant women: effects on thyroid function and Tg

**Figure 2:**
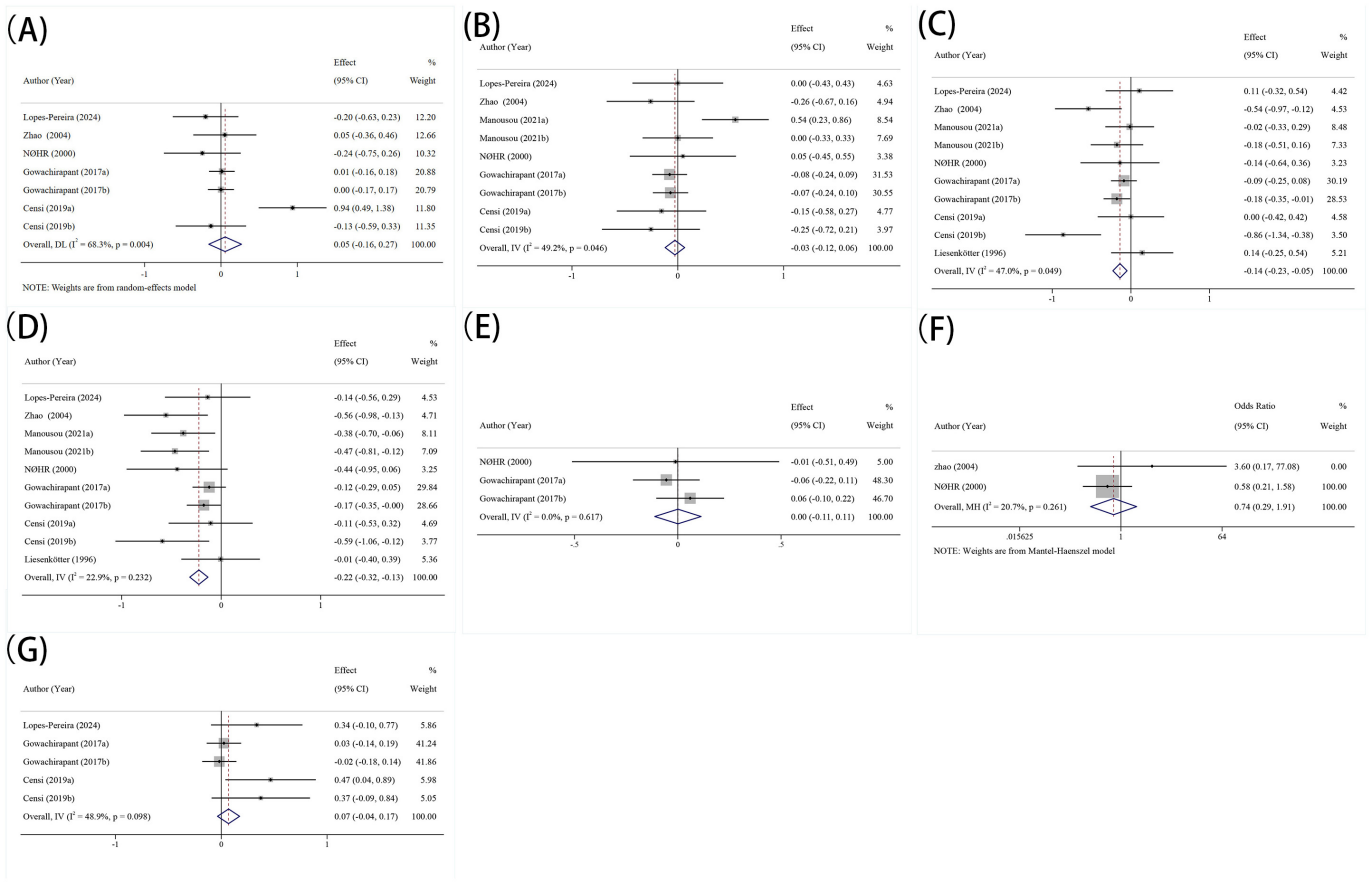
Forest plot of the effect of iodine supplementation on thyroid function in iodine-deficient pregnant women. **(A)** FT_3_; **(B)** FT_4_; **(C)** TSH; **(D)**Tg; **(E)** TPOAb; **(F)** TgAb; **(G)** TV.

The relationship between iodine supplementation for iodine-deficient pregnant women and serum FT_3_ levels is shown in Figure 2A. A total of 5 articles, encompassing 7 studies, were included. The iodine supplementation group comprised 762 pregnant women, while the control group had 714 pregnant women. The heterogeneity test indicates moderate heterogeneity (*I*^2^ = 68.3%, *Phet* = 0.004), therefore a random-effects model is adopted for meta-analysis. The meta-analysis results showed that a total of 6 studies crossed the invalid line. The pooled effect was 0.05, (95% CI: −0.16, 0.27; *Pz* = 0.615). The analysis results of iodine supplementation and serum FT_4_ levels in iodine-deficient pregnant women showed that a total of 6 articles, including 9 studies, were included. The intervention group of iodine supplementation consisted of 936 pregnant women, while the control group consisted of 891 pregnant women. The heterogeneity test indicates low heterogeneity (*I*^2^ = 49.2%, *Phet* = 0.046), therefore a fixed-effects model is adopted for pooled analysis. The results of the meta-analysis are shown in Figure 2B, with a total of 8 studies crossed the invalid line (SMD= −0.03, 95% CI: −0.12, 0.06; *Pz* = 0.561). The above results suggest a correlation between iodine supplementation in pregnant women and elevated FT_3_ levels as well as decreased FT_4_ levels, but these relationships are not statistically significant. Figure 2C illustrates the relationship between iodine supplementation and serum TSH levels in iodine-deficient pregnant women. The study revealed that a total of 7 articles, encompassing 10 studies, were included. The intervention group for iodine supplementation comprised 967 pregnant women, while the control group had 955 pregnant women. The heterogeneity test indicates low heterogeneity (*I*^2^ = 47%, *Phet* = 0.049), therefore a fixed-effects model is adopted for pooled analysis. The meta-analysis results showed that a total of 7 studies crossed the invalid line. The pooled effect was −0.14, (95% CI: −0.23, −0.05; *Pz* = 0.002). The results suggest a close statistical correlation between iodine supplementation in pregnant women and reduced TSH levels.

The relationship between iodine supplementation in iodine-deficient pregnant women and serum Tg levels is shown in Figure 2D. A total of 7 articles, including 10 studies, were included. The intervention group in the iodine supplementation group consisted of 951 pregnant women, while the control group consisted of 928 pregnant women. The heterogeneity test indicates low heterogeneity (*I*^2^ = 22.9%, *Phet* = 0.232), therefore a fixed-effects model is adopted for pooled analysis. The meta-analysis results showed that a total of 6 studies crossed the invalid lines (SMD = −0.22, 95% CI: −0.32, −0.13; *Pz* < 0.001). There is a significant statistical correlation between iodine supplementation for pregnant women and a decrease in their Tg levels.

### 3.3 Iodine supplementation in iodine-deficient pregnant women: effects on thyroid autoimmune antibodies and TV

The results of the correlation analysis between iodine supplementation for iodine-deficient pregnant women and thyroid autoantibody levels are shown in Figures 2E, F. Among them, the relationship between iodine supplementation for iodine-deficient pregnant women and TPOAb levels was included in 2 articles with 3 studies, with 617 cases in the iodine supplementation group and 610 cases in the control group. The heterogeneity test indicated no heterogeneity (*I*^2^ = 0.0%, *Phet* = 0.617), and a fixed-effects model was used for meta-analysis. The results showed that the effect sizes of the 3 studies all crossed the invalid line. The pooled effect was 0.00(95% CI: −0.11, 0.11, *Pz* = 0.992). In the analysis of the relationship between iodine supplementation and TgAb levels in iodine-deficient pregnant women, a total of 2 articles including 2 studies were included. There were 97 cases in the iodine supplementation group and 62 cases in the control group. The heterogeneity test indicated low heterogeneity (*I*^2^ = 20.7%, *Phet* = 0.261) thus a fixed-effects model is adopted for pooled analysis. The meta-analysis results showed that a total of 2 studies crossed the invalid line. The pooled effect was 0.74 (95% CI: 0.29, 1.91, *Pz* = 0.535). These results suggest that iodine supplementation did not significantly affect the levels of TPOAb and TgAb in pregnant women.

Figure 2G illustrates the relationship between iodine supplementation for iodine-deficient pregnant women and TV. A total of 3 articles, encompassing 5 studies, were included. The intervention group in the iodine supplementation group consisted of 718 pregnant women, while the control group had 697 pregnant women. The heterogeneity test indicated low heterogeneity (*I*^2^ = 48.9%, *Phet* = 0.098), therefore a fixed-effects model was employed for pooled analysis. The meta-analysis results revealed that a total of four studies crossed the invalid line. The pooled effect was 0.07 (95% CI: −0.04, 0.17; *Pz* = 0.196), but it is not statistically significant. It is suggested that iodine supplementation in pregnant women with iodine deficiency is associated with increased TV, but this relationship is not statistically significant.

### 3.4 Subgroup analysis

The above findings suggest heterogeneity in FT_3_, FT_4_, TSH, Tg and TV. To identify the sources of heterogeneity, subgroup analyses were performed (Table 3). Due to insufficient studies on TPOAb and TgAb (*n* ≤ 5), which might bias test power, subgroup analyses were not conducted for these indicators in this study. For the grouping strategy of iodine supplementation dosage, we formulated the grouping standard strictly in accordance with internationally recognized authoritative guidelines. Specifically, the American Thyroid Association (ATA) recommends a daily iodine intake of 150 μg for pregnant women, whereas the WHO suggests 250 μg/d (19, 36). On the basis of the above guideline values combined with the actual iodine intake distribution characteristics of our enrolled subjects, 200 μg/d was determined as the cut-off value for low iodine intake. Meanwhile, 300 μg/d was defined as the upper limit of high-dose group in view of the maximum iodine intake of enrolled participants. Accordingly, all subjects were divided into two subgroups: iodine intake < 200 μg/d and 200–300 μg/d.

**Table 3:** Subgroup meta-analysis of FT_3_, FT_4_, TSH, Tg, and TV.

Subgroup	No. of studies	SMD 95%CI	*Pz*	*I^2^*(%)	*Phet*
FT_3_					
**Country**					
Asia	3	0.01 (−0.11, 0.12)	0.871	0.0	0.975
Europe	4	0.09 (−0.47, 0.66)	0.745	83.6	< 0.001
**Gestation**					
First trimester	1	−0.20 (−0.63, 0.23)	0.359	NA	NA
Second trimester	2	0.45 (−0.46, 1.36)	0.329	93.2	< 0.001
Third trimester	3	−0.04 (−0.19, 0.12)	0.636	0.0	0.609
Overall trimester	1	0.05 (−0.36, 0.46)	0.809	NA	NA
**Total duration of iodine supplementation**					
At least 3 months	1	0.05 (−0.36, 0.46)	0.809	NA	NA
Everyday	4	−0.02 (−0.13, 0.09)	0.719	0.0	0.651
Until postpartum	2	0.41 (−0.64, 1.45)	0.449	90.7	0.001
**Iodine supplementation dosage**					
< 200 μg/d	3	−0.12 (−0.37, 0.14)	0.378	0.0	0.599
200–300 μg/d	4	0.16 (−0.14, 0.47)	0.295	81.8	< 0.001
**Iodine supplementation methods**					
Potassium iodide	5	0.10 (−0.17, 0.37)	0.463	77.5	0.001
Vitamin or/and mineral	2	−0.07 (−0.39, 0.25)	0.677	0.0	0.375
FT_4_					
**Country**					
Asia	3	−0.09 (−0.20, 0.03)	0.132	0.0	0.704
Europe	6	0.09 (−0.07, 0.25)	0.257	57.7	0.037
**Gestation**					
First trimester	1	0.00 (−0.43, 0.43)	1.000	NA	NA
Second trimester	3	0.03 (−0.10, 0.17)	0.638	84.0	0.002
Third trimester	4	−0.06 (−0.20, 0.07)	0.353	0.0	0.805
Overall trimester	1	−0.26 (−0.67, 0.16)	0.225	NA	NA
**Total duration of iodine supplementation**					
At least 3 months	1	−0.26 (−0.67, 0.16)	0.225	NA	NA
Everyday	6	0.00 (−0.10, 0.10)	0.950	61.3	0.024
Until postpartum	2	−0.20 (−0.51, 0.11)	0.211	0.0	0.756
**Iodine supplementation dosage**					
< 200 μg/d	5	0.12 (−0.05, 0.29)	0.164	63.5	0.027
200–300 μg/d	4	−0.09 (−0.20, 0.02)	0.113	0.0	0.884
**Iodine supplementation methods**					
potassium iodide	5	−0.08 (−0.19, 0.02)	0.125	0.0	0.938
Vitamin or/and mineral	4	0.14 (−0.04, 0.33)	0.129	71.7	0.014
TSH					
**Country**					
Asia	3	−0.16 (−0.28, −0.05)	0.005	48.3	0.144
Europe	7	−0.10 (−0.25, 0.05)	0.187	52.7	0.048
**Gestation**					
First trimester	2	0.13 (−0.16, 0.42)	0.392	0.0	0.902
Second trimester	3	−0.07 (−0.20, 0.07)	0.351	0.0	0.873
Third trimester	4	−0.23 (−0.37, −0.09)	0.001	58.2	0.067
Overall trimester	1	−0.54 (−0.97, −0.12)	0.012	NA	NA
**Total duration of iodine supplementation**					
At least 3 months	1	−0.54 (−0.97, −0.12)	0.012	NA	NA
Everyday	6	−0.11 (−0.21, −0.01)	0.027	0.0	0.819
Until postpartum	3	−0.17 (−0.42, 0.08)	0.176	81.7	0.004
**Iodine supplementation dosage**					
< 200 μg/d	5	−0.14 (−0.31, 0.03)	0.113	25.7	0.250
200–300 μg/d	5	−0.14 (−0.25, −0.03)	0.010	65.5	0.021
**Iodine supplementation methods**					
Potassium iodide	6	−0.13 (−0.23, −0.02)	0.017	60.9	0.025
Vitamin or/and mineral	4	−0.18 (−0.37, 0.00)	0.052	23.0	0.273
Tg					
**Country**					
Asia	3	−0.18 (−0.29, −0.06)	0.003	43.5	0.170
Europe	7	−0.30 (−0.45, −0.15)	< 0.001	6.5	0.378
**Gestation**					
First trimester	2	−0.06 (−0.36, 0.23)	0.662	0.0	0.662
Second trimester	3	−0.17 (−0.31, −0.03)	0.019	4.5	0.351
Third trimester	4	−0.28 (−0.42, −0.14)	< 0.001	35.4	0.200
Overall trimester	1	−0.56 (−0.98, −0.13)	0.010	NA	NA
**Total duration of iodine supplementation**					
At least 3 months	1	−0.56 (−0.98, −0.13)	0.010	NA	NA
Everyday	6	−0.21 (−0.31, −0.11)	< 0.001	7.6	0.368
Until postpartum	3	−0.20 (−0.45, 0.05)	0.113	47	0.151
**Iodine supplementation dosage**					
< 200 μg/d	5	−0.40 (−0.57, −0.23)	< 0.001	0.0	0.707
200–300 μg/d	5	−0.16 (−0.26, −0.05)	0.004	2.9	0.390
**Iodine supplementation methods**					
Potassium iodide	6	−0.16 (−0.26, −0.05)	0.003	0.0	0.531
Vitamin or/and mineral	4	−0.45 (−0.64, −0.26)	< 0.001	0.0	0.935
TV					
**Country**					
Asia	2	0.00 (−0.11, 0.12)	0.957	0.0	0.710
Europe	3	0.39 (0.14, 0.65)	0.002	0.0	0.910
**Gestation**					
First trimester	1	0.34 (−0.10, 0.77)	0.128	NA	NA
Second trimester	2	0.08 (−0.07, 0.23)	0.297	72.0	0.059
Third trimester	2	0.02 (−0.13, 0.18)	0.760	59.0	0.118
Overall trimester	NA	NA	NA	NA	NA
**Total duration of iodine supplementation**					
At least 3 months	NA	NA	NA	NA	NA
Everyday	3	0.03 (−0.09, 0.14)	0.658	11.5	0.323
Until postpartum	2	0.42 (0.11, 0.74)	0.008	0.0	0.774
**Iodine supplementation dosage**					
< 200 μg/d	1	0.34 (−0.10, 0.77)	0.128	NA	NA
200–300 μg/d	4	0.05 (−0.06, 0.16)	0.340	52.5	0.099
**Iodine supplementation methods**					
Potassium iodide	5	0.07 (−0.04, 0.17)	0.196	48.9	0.098
Vitamin or/and mineral	NA	NA	NA	NA	NA

FT_3_, free triiodothyronine; FT_4_, free thyroxine; TSH, thyroid stimulating hormone; Tg, thyroglobulin; TV, thyroid Volumes; ISM, iodine supplementation methods; TDIS, total duration of iodine supplementation;ISD: iodine supplementation dosage; CI, confidence interval; NA, not available; SMD, standardized mean difference; *Pz* , *p*-value for overall effect.; *Phet, p*-value for heterogeneity.

The FT_3_ subgroup analysis is presented in Table 3. We identified some sources of heterogeneity (*I*^2^ > 50%) in the “Europe” subgroup, the “second trimester” subgroup, the subgroup with a total duration of iodine supplementation of “iodine supplementation throughout pregnancy until postpartum”, the subgroup with an iodine supplementation dose of “200–300 μg/d”, and the subgroup with an iodine supplementation method of “potassium iodide ”. The FT_4_ analysis results (Table 3) indicate that heterogeneity (*I*^2^ > 50%) still exists in the “Europe” subgroup, the “second trimester” subgroup, “overall trimester” for total duration of iodine supplementation, “ < 200 μg/d” for iodine supplementation dose, and “ vitamins or/and minerals” for iodine supplementation method. However, we were unable to find corresponding subgroup pooled effect sizes that were statistically different from FT_3_ and FT_4_ (*Pz* > 0.05). This suggests that there is no statistically significant association between changes in serum FT_3_ and FT_4_ levels in iodine-deficient pregnant women after iodine supplementation and factors such as country, gestation, iodine supplementation method, total duration, and dosage.

The TSH subgroup analysis results (Table 3) indicate that the TSH levels of pregnant women in the “Asia” subgroup significantly decreased after iodine supplementation for iodine deficiency (SMD = −0.16; 95% CI: −0.28, −0.05; *Pz* = 0.005; *I*^2^ = 48.3%, *Phet* = 0.144). For the gestation subgroup (as there was only one study on the overall trimester, no analysis was conducted), we identified a source of partial heterogeneity, specifically, statistically significant heterogeneity was observed in the “third trimester” (SMD = −0.23; 95% CI: −0.37, −0.09; *Pz* = 0.001; *I*^2^ = 58.2%, *Phet* = 0.067). In the subgroup of total duration of iodine supplementation (as there was only one study in the “at least 3 months” subgroup, it was not analyzed), we found that in the “daily” subgroup, TSH levels in pregnant women significantly decreased after iodine supplementation for iodine deficiency (SMD = −0.11; 95%CI: −0.21, −0.01; *Pz* = 0.027; *I*^2^ = 0.0%, *Phet* = 0.819). In the subgroup with iodine supplementation doses of “200–300 μg/d”, the TSH levels of pregnant women significantly decreased after iodine supplementation for iodine deficiency (SMD = −0.14;95%CI: −0.25, −0.03; *Pz* = 0.010; *I*^2^ = 65.5%, *Phet* = 0.021). In the analysis with “potassium iodide” as the subgroup, the decrease in TSH levels among pregnant women after iodine supplementation for iodine deficiency was more significant (SMD = −0.13; 95% CI: −0.23, −0.02; *Pz* = 0.017; *I*^2^ = 60.9%, *Phet* = 0.025). Although heterogeneity (*I*^2^ > 50%) was observed in the “Europe” subgroup and the subgroup with a total iodine supplementation duration of “iodine supplementation throughout pregnancy until postpartum”, no statistically significant pooled effect size was found (*Pz* > 0.05).

The results of the Tg subgroup analysis are presented in Table 3. In various country subgroups (Asia and Europe), subgroups with different iodine supplementation doses (< 200 μg/d and 200–300 μg/d), and subgroups with different iodine supplementation methods (potassium iodide and vitamins or/and mineral), the Tg levels of iodine-deficient pregnant women were significantly reduced (*Pz* < 0.05). For the gestation subgroup analysis (not analyzed due to only one study covering the overall trimester), the results showed that heterogeneity persisted in the “second trimester” and “third trimester” subgroups (second trimester subgroups: *I*^2^ =4.5%, *Phet* = 0.351; third trimester subgroups: *I*^2^ = 35.4%, *Phet* = 0.200). Furthermore, after iodine supplementation for pregnant women with iodine deficiency in the second and third trimester subgroups, Tg levels significantly decreased (second trimester subgroups: SMD = −0.17; 95% CI: −0.31, −0.03; *Pz* = 0.019; third trimester subgroups: SMD = −0.28; 95% CI: −0.42, −0.14; *Pz* < 0,001). In the subgroup with a total duration of iodine supplementation (only one study with an iodine supplementation duration of at least 3 months was not analyzed), we found a significant decrease in Tg levels among iodine-deficient pregnant women in the “daily” iodine supplementation subgroup (SMD = −0.21; 95% CI: −0.31, −0.11; *Pz* < 0.001; *I*^2^ = 7.6%, *Phet* = 0.368).

The TV subgroup analysis results showed that in the “Europe” subgroup, the thyroid volume significantly increased after iodine supplementation for iodine deficiency in pregnant women (SMD = 0.39; 95% CI: 0.14, 0.65; *Pz* = 0.002; *I*^2^ = 0.0%, *Phet* = 0.910). In the subgroup of total duration of iodine supplementation (there was only one study in the subgroup of “at least 3 months”, which was not analyzed), the results showed that the TV of iodine deficient pregnant women in the subgroup of “iodine supplementation throughout pregnancy until postpartum” increased significantly (SMD = 0.42; 95%CI: 0.11,0.74; *Pz* = 0.008; *I*^2^ = 0.0%, *Phet* = 0.774). In the subgroup of “iodine supplementation methods”, we did not analyze them because the iodine supplementation methods were all “potassium iodide ”. In addition, we found that there was still high heterogeneity (*I*^2^ > 50%) in the “second trimester” subgroup, the “third trimester” subgroup, and the “200–300 μg/d” subgroup of iodine supplementation dose, but we failed to obtain a statistically significant pooled effect (*Pz* > 0.05).

### 3.5 Sensitivity analyses and publication bias

**Figure 3:**
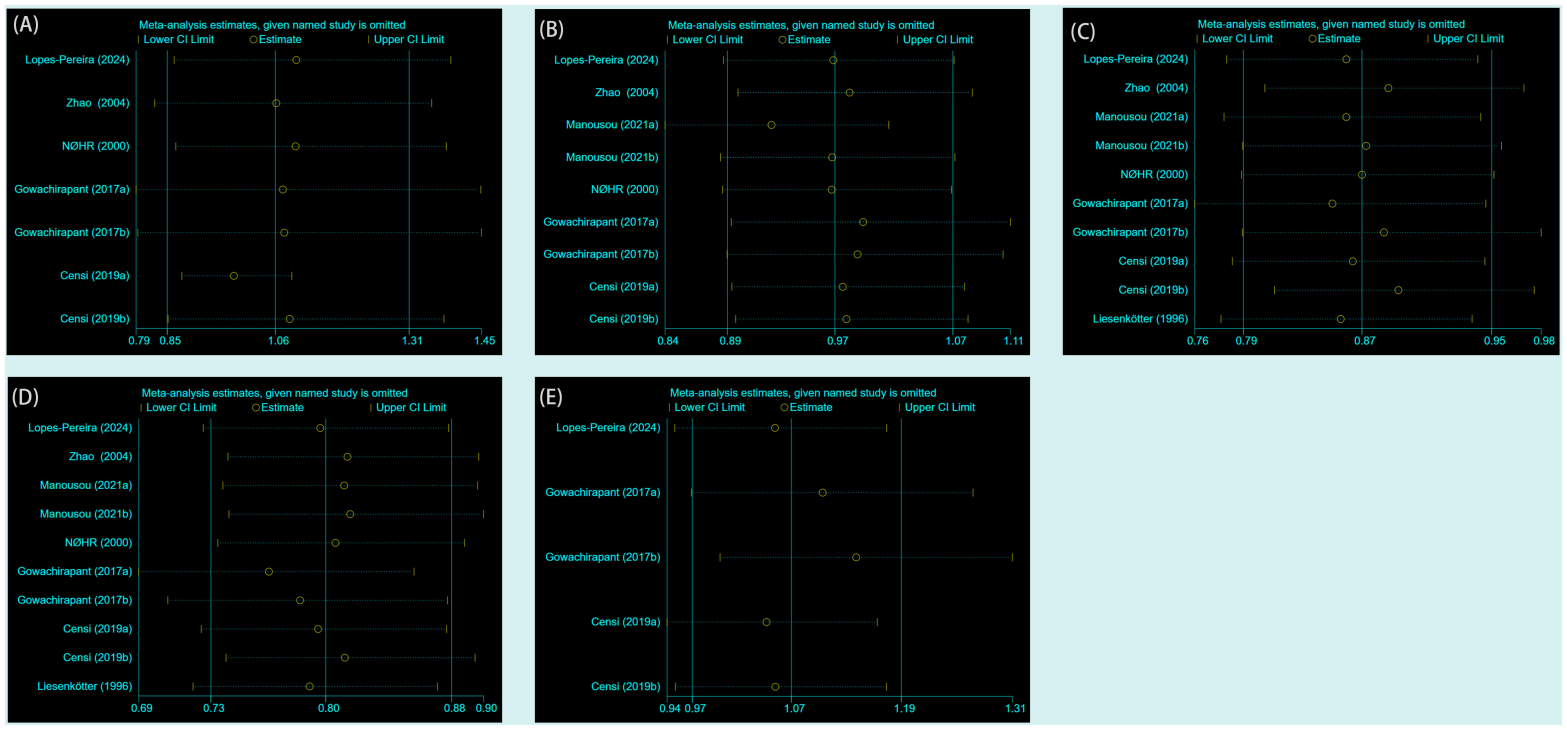
The result of sensitivity analysis. **(A)** FT_3_; **(B)** FT_4_; **(C)** TSH; **(D)**Tg; **(E)** TV.

We conducted sensitivity analysis on FT_3_, FT_4_, TSH, Tg and TV, and the meta-analysis results were generally stable after eliminating each individual study (Figure 3). Egger's test showed that only TV had publication bias (FT_3_: *P* = 0.755; FT_4_: *P* = 0.830; TSH: *P* = 0.636; Tg: *P* = 0.100; TV: *P* = 0.005). Subsequently, the Trim-and-Fill method was used for quantitative correction of TV. The pooled effect size (SMD) of the original fixed-effect model was 0.07 (95% CI: −0.04, 0.17; *Pz* = 0.196). The Trim-and-Fill method estimated the absence of 2 small-sample negative studies, with the corrected pooled effect size (SMD) at 0.03 (95% CI; −0.07, 0.12; *Pz* = 0.621). After correction, the pooled effect size (SMD) decreased and the 95% CI was closer to the null value, with no change in statistical significance (all *Pz* > 0.05). This suggests publication bias had little impact on the results, which were relatively robust. Due to fewer than 5 studies on thyroid antibodies TPOAb and TgAb precluding reliable results, sensitivity analysis and publication bias testing were not performed.

## 4 Discussion

As an essential trace element for thyroid hormone synthesis, an adequate iodine supply during pregnancy plays an irreplaceable role in maintaining maternal thyroid function homeostasis and supporting normal fetal neurointellectual development (37). Based on 7 articles, this meta-analysis systematically evaluated the effects of iodine supplementation interventions on thyroid function, related indicators, and secondary outcome indicators in iodine-deficient pregnant women.

In this study, we found that iodine supplementation had no significant effect on serum FT_3_ and FT_4_ levels in iodine-deficient pregnant women, and subgroup analyses also revealed no significant regulatory effect of iodine supplementation on these levels under any stratified conditions (38). Moreover, existing literature has shown that iodine supplementation primarily acts on the compensatory regulatory process of the thyroid gland to alleviate thyroid functional stress without disrupting the physiological homeostasis of free thyroid hormones (39). This also suggests that the findings of this study are consistent with the synthesis and regulatory mechanisms of thyroid hormones, as well as the physiological adaptive characteristics of pregnant women during gestation.

TSH is a core indicator reflecting the regulatory status of thyroid function, while Tg primarily reflects thyroid structure and proliferative degree. This study found that iodine supplementation in iodine-deficient pregnant women significantly reduced serum TSH and Tg levels, suggesting that iodine supplementation can not only effectively restore maternal thyroid function but also inhibit compensatory hyperplasia. This comprehensively protects thyroid health during pregnancy from both functional and structural perspectives, reduces the risk of abnormal thyroid function and nodules in gestation, and is of great significance for improving maternal and fetal outcomes. This is also consistent with the findings of Pedersen et al., who observed a significant increase in TSH and Tg levels in pregnant women without iodine supplementation during gestation (40). This clearly reflects that, under mild iodine deficiency, maternal thyroid glands exhibit a marked stress response due to increased physiological demands of the thyroid during pregnancy, leading to compensatory changes in thyroid function. However, some studies have suggested that daily iodine supplementation of 200 μg in pregnant women can significantly reduce TSH and Tg levels and alleviate thyroid burden, which clearly confirms the effectiveness of this dosage in improving thyroid stress status in pregnant women with mild iodine deficiency during gestation (40). Nevertheless, results of TV analysis showed that iodine supplementation only led to a non-significant increasing trend in TV among iodine-deficient pregnant women, which may be attributed to the longer intervention duration required for changes in thyroid volume (41). Additionally, research has indicated that pregnant women with long-term iodine deficiency who initiated iodine supplementation before pregnancy had a more significant effect on improving TV and preventing goiter compared with those who only started supplementation during pregnancy (42).

In the present study, subgroup analyses were performed to explore the sources of heterogeneity, and some sources of heterogeneity were identified. Interestingly, the effect of iodine supplementation in iodine-deficient pregnant women on significantly reducing serum TSH levels was more pronounced in subgroups of Asian populations, third trimester of pregnancy, daily iodine supplementation during pregnancy, potassium iodide as the supplementation form, and iodine dosage of 200–300 μg/d. This may be associated with the high burden of iodine deficiency in Asia, increased iodine demand in the third trimester, and more prominent thyroid compensation (43). A study conducted in Harbin, China showed that urinary iodine concentration in pregnant women gradually decreased with advancing gestational age, and was significantly lower in the third trimester than in the first trimester. At the same time, the detection rate of thyroid dysfunction is higher in late pregnancy, indicating that maternal thyroid compensation function is more pronounced when the iodine burden is heavier (44). However, our research does not deny the importance of continuous iodine supplementation in early and mid-pregnancy. In short, we should pay attention to the foundation of full iodine nutrition, especially strengthen iodine nutrition monitoring and personalized supplementation in late pregnancy, in order to more accurately maintain normal maternal thyroid function. Pregnant women have a significant increase in iodine demand, and elevated TSH levels are key manifestations of thyroid dysfunction caused by iodine deficiency. Potassium iodide tablets have stable iodine content and high bioavailability, which can more accurately control dosage compared to dietary iodine supplementation. This is the core reason why they have more advantages in regulating TSH levels (45). Meanwhile, the recommended gestational iodine doses by the ATA and the WHO are highly consistent with the dose range in the present study. The ATA recommends iodine supplement of 150 μg/d for pregnant women, with an upper limit of 500 μg/d for total iodine intake, while the WHO recommends iodine intake of 250 μg/d. Such doses can maintain maternal thyroid function, meet fetal developmental requirements, and ensure the safety of iodine supplementation (19, 36). In addition, several noteworthy findings emerged from the subgroup analyses regarding the effect of iodine supplementation on Tg levels in iodine-deficient pregnant women. We found that daily regular iodine supplementation during pregnancy significantly reduced serum thyroglobulin (Tg) levels, regardless of whether participants were Asia or Europe, in the second or third trimester, with a supplementation dosage of < 200 μg/d or 200–300 μg/d, and whether supplementation was provided as potassium iodide tablets or vitamins or mineral preparations. This result suggests that the lowering effect of iodine supplementation on Tg levels in pregnant women with iodine deficiency is widely consistent, and is not significantly affected by population location, pregnancy cycle, reasonable iodine supplementation dose, or iodine supplementation method. The core reasons are closely related to the biological characteristics of Tg, physiological commonalities of iodine deficient pregnant women, and the core mechanism of iodine supplementation. However, TV subgroup analysis showed that TV levels were significantly elevated only in the European population and iodine deficient pregnant women who received iodine supplementation throughout pregnancy until postpartum. This phenomenon may be related to European dietary habits and the physiological adaptation of the thyroid gland to pregnancy and lactation (46, 47). Some studies suggest that when iodine deficient pregnant women continue to receive iodine supplementation from pregnancy to postpartum, the increase in thyroid volume is mainly driven by physiological stimuli during pregnancy, rather than compensatory hypertrophy caused solely by iodine deficiency (47). Therefore, even if iodine supplementation has corrected iodine deficiency, the thyroid gland can still experience volume increase under the action of pregnancy related hormones. However, the above conclusion is based on subgroup analysis, and due to the limited number of studies within each subgroup, the interpretation of the results needs to be cautious.

Regarding autoimmunity, this study found no significant effect of iodine supplementation on TPOAb and TgAb levels in iodine-deficient pregnant women. This result is consistent with the conclusions of several previous studies on iodine nutrition intervention during pregnancy (48–50). Konrade et al. observed that neither the level of iodine supplementation nor the use of iodized salt increased the risk of elevated TPOAb, and iodine supplementation at the recommended dose would not induce thyroid autoimmune reactions (49). In contrast, a study conducted in Xinjiang, China suggested that there were no significant differences in the positive rate of TPOAb, the positive rate of TgAb, or the combined positive rate of TPOAb and TgAb among pregnant women in different trimesters across four regions of Xinjiang (50). As is well known, the occurrence of thyroid autoimmunity is mainly driven by factors such as innate immune susceptibility and pre-existing immune disorders before pregnancy, as well as immune cell subpopulation imbalance (51). The pathological damage target of iodine deficiency during pregnancy focuses on the thyroid hormone synthesis cycle, rather than directly inducing thyroid follicular cell destruction and releasing TPO/Tg self-antigens. Therefore, iodine supplementation can only correct iodine nutrition status and restore hormone synthesis function, but cannot reverse the existing autoimmune response. This may be the core reason why iodine supplementation does not affect antibody levels (52). At the same time, the iodine supplementation regimens included in this study all comply with the recommended reasonable doses for pregnancy by the ATA and WHO, and there are no cases of excessive iodine exposure. Previous studies have confirmed that only excessive iodine can damage follicular cells and expose self- antigens through oxidative stress, thereby affecting self-antibody levels (53), which further explains the rationality of the results in this study that iodine supplementation has no significant effect on TPOAb and TgAb.

Overall, this study identified differences in the effectiveness of iodine supplementation for iodine-deficient pregnant women under different iodine intervention strategies, and identified effective scenarios for regulating relevant iodine supplementation indicators. This provides important evidence for iodine supplementation intervention strategies in Asia and during the late pregnancy period.

There are some limitations in this study. First, this study mainly focused on changes in thyroid function concentrations, but the follow-up duration was relatively short, failing to fully assess the impact of iodine supplementation on maternal outcomes (such as pregnancy complications) and the long-term neurointellectual development of infants. Second, although subgroup analyses suggested that iodine supplementation was more effective in Asian pregnant women and those in the third trimester, due to the limitations of the burden of iodine deficiency and dietary differences within the Asian region, caution should be exercised when extrapolating this conclusion to other regions. Third, subgroup analyses were *post-hoc* stratified analyses; after stratification, the number of studies and sample size in each subgroup decreased, leading to reduced statistical power. In the future, it is necessary to further expand the sample size to explore the sources of heterogeneity. Fourth, differences existed in the detection kits and laboratory reference ranges across the included studies. Although SMD was adopted for unified quantitative measurement, methodological heterogeneity could not be eliminated, which may potentially affect the robustness of the results. Finally, the analysis of autoimmune indicators (TPOAb, TgAb) was limited by the insufficient number of studies, making it impossible to conduct more in-depth subgroup discussions, which may affect the comprehensive assessment of the risk of iodine excess. Therefore, further studies are needed to confirm these results.

## 5 Conclusions

Standardized iodine supplementation in iodine-deficient pregnant women can significantly reduce serum TSH and Tg levels, among which the improvement in TSH is more pronounced in Asian populations, those in the third trimester of pregnancy, and those receiving 200–300 μg of potassium iodide daily. A significant increase in TV was only observed in European populations and iodine-deficient pregnant women who received continuous iodine supplementation from pregnancy to postpartum. This study found no significant effect of iodine supplementation on serum FT_3_, FT_4_, TPOAb, or TgAb levels in iodine-deficient pregnant women. This study advances the theory of iodine nutrition regulation during pregnancy, clarifies the stratified characteristics of iodine supplementation effects, provides evidence-based reference for the precise clinical intervention of iodine deficiency during pregnancy, and simultaneously offers important theoretical and clinical value for protecting maternal and fetal health and avoiding the risks of iodine supplementation.

## Data Availability

The original contributions presented in the study are included in the article/supplementary material, further inquiries can be directed to the corresponding authors.
